# Minimally Invasive Spine Surgery for Kyphoscoliosis in a Patient With Parkinson’s Disease: A Case Report

**DOI:** 10.7759/cureus.39397

**Published:** 2023-05-23

**Authors:** Ioannis Polythodorakis, Alexandros Brotis, Charalampos Charitidis, Vasilios Lycomitros, Iason Liveris, Kostantinos Paterakis

**Affiliations:** 1 Neurosurgery, Henry Dunant Hospital, Athens, GRC; 2 Neurosurgery, General University Hospital of Larisa, Larisa, GRC; 3 Orthopaedic Surgery, Henry Dunant Hospital, Athens, GRC; 4 Neurological Surgery, Henry Dunant Hospital, Athens, GRC; 5 Neurosurgery, Universtity Hospital of Larissa, Larissa, GRC

**Keywords:** minimally invasive surgery, parkinson's disease, lumbar, kyphoscoliosis, deformity

## Abstract

The surgical treatment for severe deformity correction in patients with Parkinson's disease (PD) is usually challenging, requiring lengthy fusions, and with a high risk of postoperative complications. We present a patient with severe kyphoscoliosis and medical history of PD undergoing minimally invasive surgical deformity correction.

A 75-year-old female with a 10-year history of medically controlled PD presented at our hospital's outpatient reporting progressive postural changes during the last two years and a half. On clinical examination, we recognized severe kyphoscoliosis associated with Pisa deformity, in the absence of any neurological manifestations. On the initial x-rays, the coronal angulation was 56° in the lumbar area with a significant lateral shift of the trunk, while the right ribs were close to the iliac crest. The patient underwent deformity correction with percutaneous pedicle screws from T5 to S1, a percutaneous transverse process hooks at T5, and transforaminal lumbar interbody fusion at L5-S1. The total duration of the operation was seven hours, and the estimated blood loss was approximately 300 mL. Clinically, the patient's posture improved significantly, alleviating any preoperative compensatory mechanisms such as knee flexion. The postoperative x-rays revealed a very satisfying correction in both the coronal and sagittal planes (20.1 degrees and 26.6 degrees, respectively).

Our current case report showed that MIS constitutes a viable alternative for deformity correction in selected patients with PD as part of a multidisciplinary approach. Proper patient selection requires a detailed medical history and a complete neurological and musculoskeletal examination by a dedicated healthcare provider.

## Introduction

Numerous neuromuscular diseases, including Parkinson's disease (PD), multiple sclerosis, cerebral palsy, and various myopathies, are associated with spinal deformities in the coronal and sagittal planes. PD is the most common neurodegenerative disease, affecting approximately 1%-2% of the population over 65 years of age [1]. Degeneration is confined to the basal ganglia, and results in various movement disorders manifested by resting tremor, muscle rigidity, and bradykinesia with a shuffling gait, increasingly forward posture, and loss of facial expression.

In about one-third of PD cases, motor manifestations are complicated by postural abnormalities [2,3]. These include antecollis, camptocormia, Pisa syndrome, kyphosis with or without scoliosis, and striatal hands and toes. The prevalence of camptocormia and lateral deformities ranges from 3% to 17.6% and 8.5% to 60%, respectively [3]. The optimal treatment for deformity correction in selected patients is usually surgical intervention, including lengthy fusions associated with an extensive operative field, significant blood loss, prolonged anesthesia, and a high risk of postoperative complications [4,5].

To our knowledge, minimally invasive spine surgery (MISS) has yet to be studied adequately in kyphoscoliosis correction, particularly in managing patients with neurodegenerative disorders. Herein, we present the case of a 75-year-old female patient with severe kyphoscoliosis and medical history of PD requiring MISS. Furthermore, we discuss several radiological and clinical challenges during the treatment of this patient.

## Case presentation

A 75-year-old female with a ten-year history of medically controlled PD presented at our hospital's outpatient reporting progressive postural changes during the last two years and a half. On clinical examination, we recognized severe kyphoscoliosis associated with PISA deformity. There were no significant manifestations from the neurological examination, including motor or sensory disturbances from the long tracts and cerebellum. The postural changes resulted in significant disability in daily activities, particularly self-care and management.

**Figure 1:**
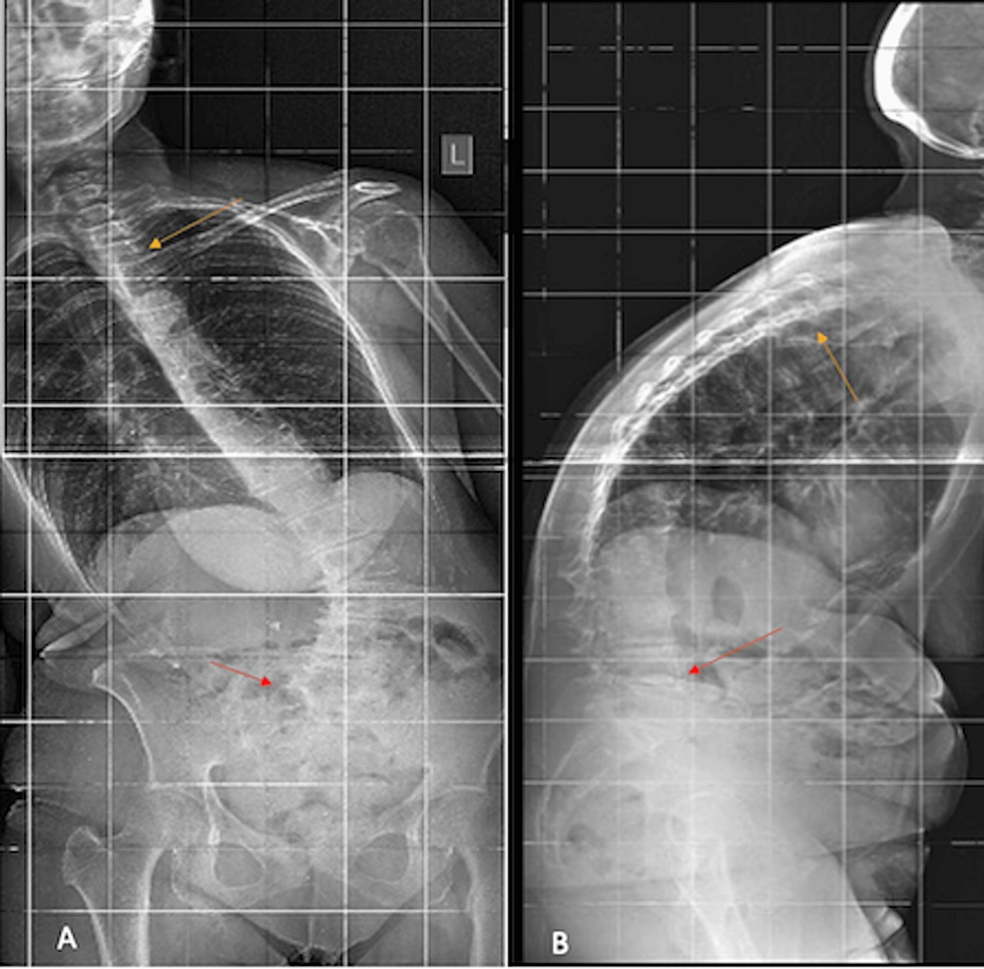
The preoperative anteroposterior (A) and lateral (B) radiographs of a 75-year-old patient with Parkinson's disease. The images depicted severe kyphoscoliosis and PISA syndrome. The yellow and red arrows show the tip and base of the curvature, respectively.

On the initial x-rays, the coronal angulation was 56° in the lumbar area with a significant lateral shift of the trunk, while the right ribs were close to the iliac crest (Figures 1A, 1B). According to the Nash-Moe scale, the apical vertebral rotation for L3 was as high as grade four [6]. At the same time, the pelvic obliquity for the L5 and S1 vertebrae was minimal, whereas the lumbar sagittal alignment was +12° with significant overall sagittal imbalance. According to the Lonstein classification, scoliosis was graded as type 2C.

The patient underwent deformity correction with percutaneous pedicle screws from T5 to S1, percutaneous transverse process hooks at T5, and transforaminal lumbar interbody fusion (TLIF) at L5-S1. The correction was completed using in situ bending in the upper thoracic spine and typical maneuvers. All interventions were under neuromonitoring control using sensory and motor-evoked potentials without any changes throughout the operation. The total duration of the operation was seven hours, and the estimated blood loss was approximately 300 mL. The patient received one unit of packed red blood cells intra-operatively and was successfully extubated at the end of the procedure. She stayed in the intensive care unit over the night and ambulated on the second postoperative day. The hospital stay was four days.

**Figure 2:**
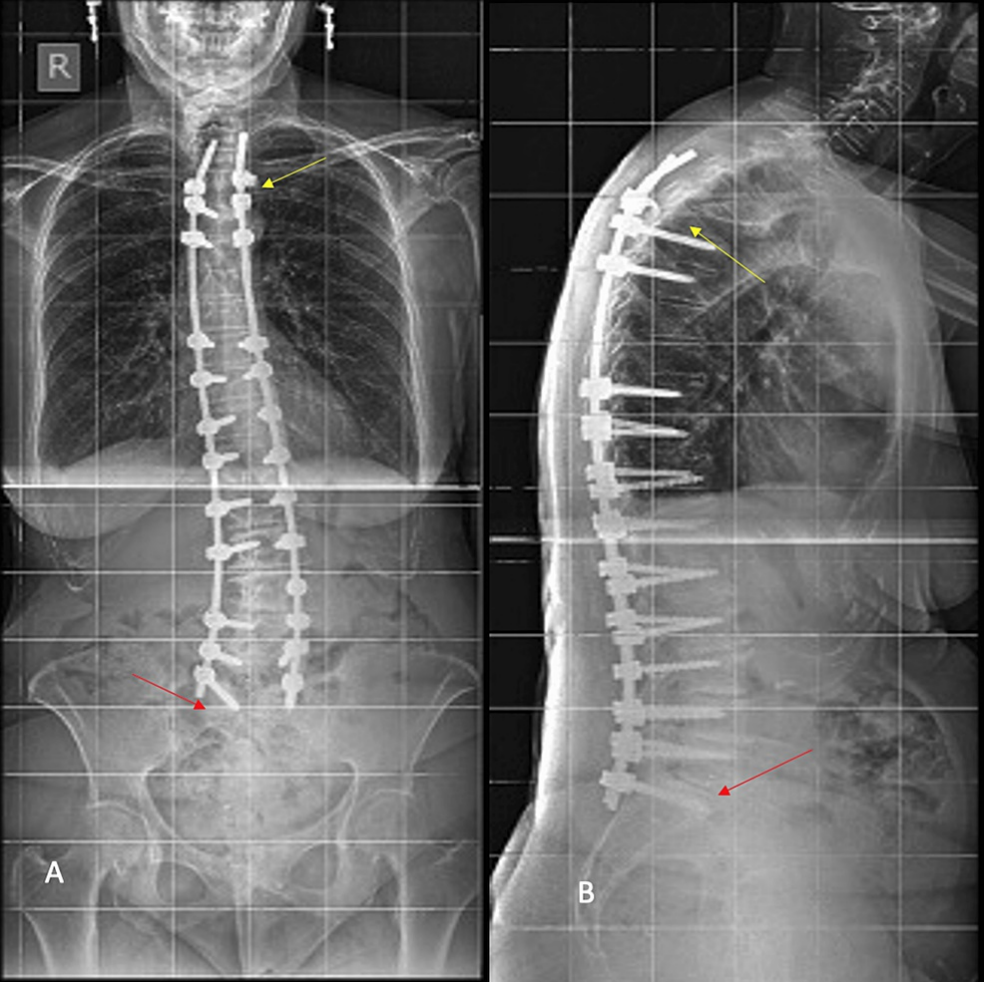
Postoperative anteroposterior (A) and lateral (B) radiographs. The yellow and red arrows show the tip of base of the curvature. The MISS approach helped to improve the patient's spinal alignment.

The postoperative x-rays revealed a very satisfying correction in the coronal and sagittal planes (20.1° and 26.6°, respectively), as depicted in Figures 2A, 2B.

Clinically, the patient's posture improved significantly, alleviating preoperative compensatory mechanisms such as knee flexion. She continued with personal everyday physiotherapy sessions at home for six weeks and then every two days for the next three months. Her walking ability and posture improved during those first six months as her muscles became stronger. Therefore, the patient's health-related quality of life was improved as well.

## Discussion

Our current case report showed that MISS constitutes a viable alternative for deformity correction in selected patients with PD. Proper patient selection requires a detailed medical history, a complete neurological and musculoskeletal examination by a dedicated healthcare provider, and advanced imaging for the apprehension of spinal alignment. As with open surgery, we also showed that proper control of PD clinical manifestations is essential before considering surgery. MIS could be associated with shorter surgical hours, smaller surgical fields, minimal blood loss, and swift recovery.

The patient with PD always constitutes a challenge to the spinal surgeon due to the increased loads and stress to the axial skeleton from the postural and motor manifestations of the disease. The extra load on the spine results in high rates of screw pullout and deformity progression, including proximal junction kyphosis, necessitating frequent surgical revisions [5-7]. Deep brain stimulation should be considered in cases of drug-resistant PD. Moreover, patients with PD frequently have osteoporosis and poor bone quality, which is partly responsible for the high pullout rate and construct failure [8]. In addition, PD is commonly associated with psychological distress and prolonged postoperative lack of motivation to mobilize, which further aggravates the overall patient functionality [7-9]. Therefore, early mobilization is the most critical target to be achieved.

Regarding the spinal alignment, Bissolotti et al. analyzed several spinopelvic parameters in a PD population and noted a reduced sacral slope and an increased pelvic tilt, representing the increased pelvic retroversion to compensate for the sagittal plane deformity and maintain an upright posture [10]. Likewise, Oh et al. evaluated the incidence of sagittal malalignment in a consecutive series of PD patients. Forty-two percent of PD patients had a positive sagittal balance with a sagittal vertical axis (SVA) greater than 50 mm [11]. Moreover, 51% of PD patients had a substantial spinopelvic mismatch, measured by the difference between pelvic incidence (PI) and lumbar lordosis (LL). At the same time, the severity of PD affected the ability to compensate using pelvic retroversion [11].

Careful patient selection, proper preoperative planning, and setting achievable goals are paramount. A thorough clinical examination, assessing the patient's posture, muscle tone, gait pattern, motor power, sensation, and reflexes, is necessary for the proper patient selection and surgical planning. The posteroanterior and lateral whole-body standing radiographs constitute the gold standard for preoperative imaging and deformity visualization in the coronal and sagittal planes. We can measure the Cobb angles, cervical lordosis, thoracic kyphosis, LL, SVA, PI, pelvic tilt, and sacral slope. The novel digital, biplanar, x-ray imaging acquisition system allows a quick assessment of the entire skeleton in a standing, weight-bearing position with a significant decrease in radiation dose compared with conventional or other digital radiography systems. A CT scan of the spine can be obtained when bony anatomy questions arise, which is a usual case in older age with many spurs and osteophytes. A whole spine MRI scan should be performed pre-operatively to exclude any other underlying pathology and to decide on the levels needing decompression in case of radicular pain or myelopathy.

The surgical goal should aim for optimal correction of the alignment parameters, which means a pelvic tilt < 25°, a C7-S1 SVA < 50 mm, and the PI minus the LL < 10° [12-14]. It is common ground for many spinal surgeons that a less aggressive correction should be considered to reduce the risk of the pullout, proximal junctional kyphosis, and instrumentation failure [7]. Traditionally, these corrections require extensive surgical exposure, associated with several disadvantages, including increased blood loss, prolonged anesthesia, and delayed recovery. MISS techniques are frequently associated with minor muscle trauma and intraoperative bleeding, and less pain which, in turn, results in shorter hospitalization and recovery. At the same time, preservation of the posterior tension band throughout the whole spine may minimize the rate of screw pullout and proximal junctional kyphosis, the most common complication in PD patients.

On the contrary, MISS for deformity correction in PD patients is very demanding technically. A long learning curve starts with applying MISS for short fusion, moving on to MISS for deformity correction, and then applying it to PD patients. Surgical times may be longer initially, and spinal surgeons who specialize in MISS should perform the more demanding cases.

Limitations

The current case report has crucial limitations since it constitutes retrospective and underpowered evidence. Our results should be read with caution, as they represent the experience of a single group of surgeons on a representative case. Further larger, high-quality studies are required to establish MISS's efficacy, safety, and cost-effectiveness for deformity correction in PD. Moreover, we must study the long-term complications associated with MISS in this specific patient subgroup, particularly the revision surgery rates. We believe that complications such as screw pullout and deformity progression are independent of the surgical approach, but are related to the management of PD itself.

## Conclusions

Our current case report showed that MISS constitutes a viable alternative for deformity correction in selected patients with PD as part of a multidisciplinary approach. Proper patient selection requires a detailed medical history, a complete neurological and musculoskeletal examination by a dedicated healthcare provider, and advanced imaging for the apprehension of spinal alignment. As with open surgery, we also showed that proper control of PD clinical manifestations is essential before considering surgery. MISS could be associated with shorter surgical hours, smaller surgical fields, minimal blood loss, and swift recovery.
